# From structure to function: unveiling the structure of the Na_v_ channel–toxin complex

**DOI:** 10.1093/nsr/nwy090

**Published:** 2018-09-14

**Authors:** Zhuan Zhou, Zuying Chai, Changhe Wang

**Affiliations:** 1 Institute of Molecular Medicine and Peking-Tsinghua Center for Life Sciences and PKU-IDG/McGovern Institute for Brain Research, Peking University, China; 2 School of Life Science and Technology and Frontier Institute of Science and Technology, Xi’an Jiaotong University, China

Voltage-gated sodium channels (Na_v_) are essential for the generation and conduction of the action potentials in excitable cells, including neurons, endocrine cells and muscles. The sodium current was first recorded and mathematically described by the Hodgkin–Huxley model in the 1950s [1], which predicted both the existence of a voltage sensor and gating currents. It took three decades for the first clone of the central pore-forming α subunit cDNA to be obtained by the Numa group [2], which was followed by structure–function and mutational studies on the voltage-dependent gating mechanism, Na^+^ selectivity and the general structure of Na_v_ channels. The first 3D crystal structure of a prokaryotic Na^+^ channel was reported by the Catterall and Zheng laboratories in 2011. The Yan and King laboratories presented three high-resolution cryo-electron microscopy (EM) structures (2.6–3.2 Å) of the eukaryotic Na_v_ channel, Na_v_PaS from American cockroach, in complex with three animal toxins [3].

**Figure 1:**
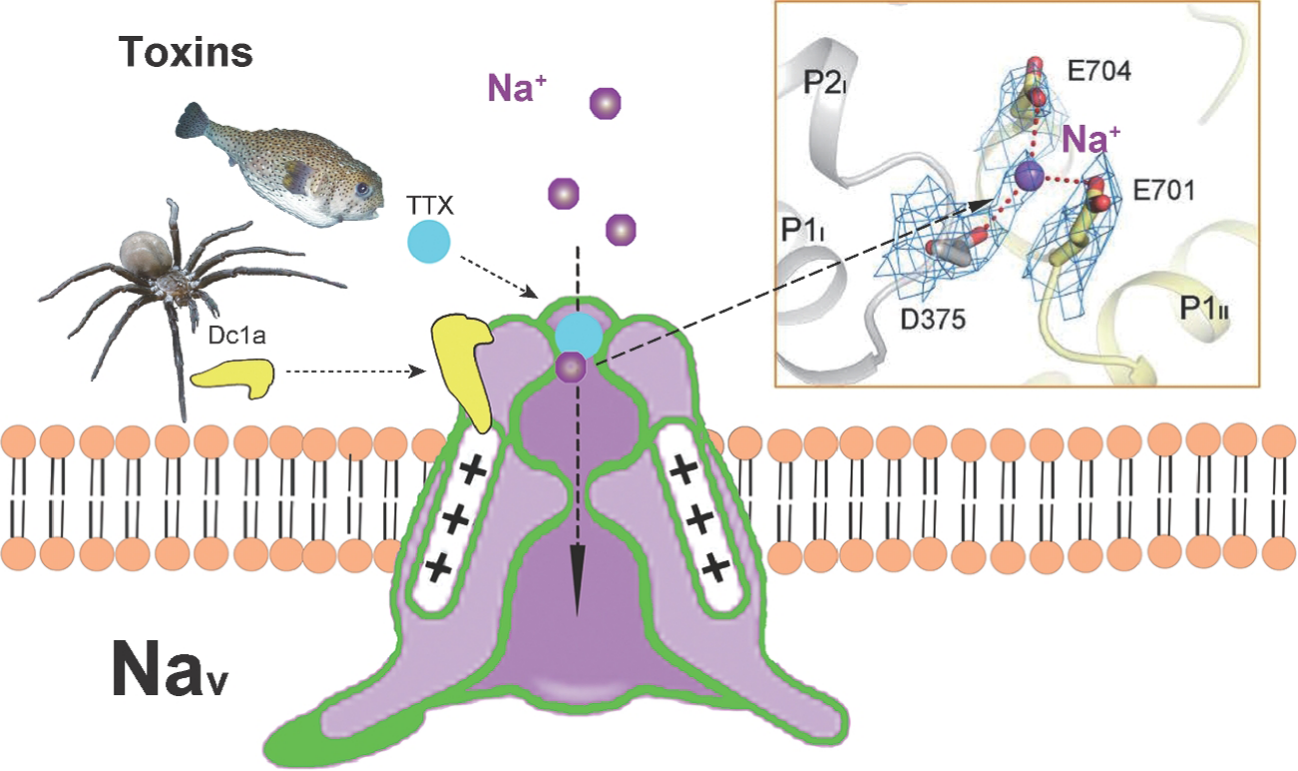
A cartoon of the Na_v_-toxin structure for how the two native toxins regulate the Na^+^ channel's function. The cryo-EM structures of the Na_v_-Dc1a-TTX/STX complexes reveal the intermolecular interactions between a Na_v_ channel and two animal toxins, and indicate a possible Na^+^ binding site in the selectivity filter. Inset (top right) is from Shen *et al*. [3].

This work is a landmark for structure–function studies of a representative voltage-gated channel interacting with toxins. Functional Na_v_ channels comprise a central pore-forming α-subunit and one to two auxiliary β subunits. The principal channel-forming α-subunit is a large polypeptide that folds into four homologous domains (I–IV) linked by three loops, each domain containing six α-helical transmembrane segments (S1–S6). The S1–S4 segments serve as the voltage-sensing module while the S5–S6 segments constitute the central ion-conducting pore module. Interestingly, Na_v_ channels are the primary and specific targets of neurotoxins from venomous organisms, which are classified as gating modifiers, such as the spider toxin Dc1a, and pore blockers represented by tetrodotoxin (TTX) and saxitoxin (STX). TTX is among the first neurotoxins that were identified as being extremely specific for Na_v_, and has played a distinct role in the ion channel research area. Impressively, the authors revealed the detailed interaction of Na_v_PaS with TTX and STX by using high-resolution cryo-EM structures. TTX/STX stabilizes at the outer vestibule through an extensive network of electrostatic interactions, effectively blocking the entrance of Na^+^ to the selectivity filter. The structure of the Na_v_PaS-Dc1a complex not only confirms the trapping mechanism between the voltage-sensing domain VSD_II_ and this gating modifier toxin, but also shows the specific interactions of Dc1a with both the reported VSD_II_ domain and the unexpected pore domain. The authors also identified a bound Na^+^ ion in the Na_v_–toxin complex and defined three acidic residues (DEE) in the selectivity filter region as a favoured binding site for Na^+^, which is very important information for determining the ion pathway within the channel (Fig. 1).

Mutations in Na_v_ genes are linked with epilepsy, cardiac arrhythmia, neuropathic pain and other pathological conditions, making them important therapeutic targets for pharmaceutical intervention. Although the selective modulation of Na_v_ by animal toxins has helped the development of Na_v_ channel drugs, this approach has been impeded by a lack of structural information on the toxin–channel intermolecular interactions.

The voltage-sensing module is a common structure shared not only by all types of voltage-gated Na^+^, Ca^2+^ and K^+^ channels, but also by at least two non-channel proteins (complex), the voltage-sensor-containing phosphatase [4] and the Ca^2+^-independent but voltage-dependent protein complex for exocytosis [5]. Thus, the high-resolution structure of Na_v_-Dc1a-TTX/STX sheds light on voltage sensors involved in multiple areas including ion channels, intracellular phosphorylation and neural secretion. Following this insect 3D structure of the Na_v_-toxin complex, many future studies are needed, such as 3D-Na_v_ structure in mammalian cells, which may offer insights into TTX-sensitive versus -insensitive Na_v_ channels, as well as their representative mutations in human patients.
